# Evidence from an Applied Research Health Question (AHRQ): Physician-prescribed medications to children for oral health issues

**DOI:** 10.23889/ijpds.v9i5.2851

**Published:** 2024-09-18

**Authors:** Samantha Morais, Diana An, Anna Durbin, Khai-Nhu Zweig, Mina Tadrous, Lesley Plumptre

**Affiliations:** 1 ICES; 2 Li Ka Shing Knowledge Institute, St. Michael’s Hospital, Unity Health Toronto; 3 Dental & Oral Health Services, Toronto Public Health; 4 Women’s College Hospital; 5 Leslie Dan Faculty of Pharmacy, University of Toronto

## Objectives

To understand changes in prescription patterns over time, and support dental and oral health program planning, Toronto Public Health issued an AHRQ request to collect information on physician-prescribed medications following an oral health-related incident.

## Approach

An algorithm was created to identify oral health-related incidents among children and youth aged up to 17 years old in Ontario and Toronto during fiscal years 2013 to 2022. The algorithm used provincial data on physician visits, emergency department visits, inpatient hospitalizations, or day surgeries to define ‘dental-related incidents’. Physician-prescribed medications (e.g., chlorhexidine, opioids, benzodiazepines, antibiotics, NSAIDs and others) within seven days of a dental-related incident were recorded using Ontario Drug Benefit Claims and the Narcotics Monitoring System.

## Results

Of 522,674 dental-related incidents in Ontario between 1 April 2013 and 31 March 2023, 9.2% had physician-prescribed medications (versus 8.6% of 90,810 dental-related incidents in Toronto). The proportion of incidents with prescriptions increased from 6.4% in 2013 to 10.7% in 2022 in Ontario, and from 5.9% to 10.3% in Toronto. The most prescribed medication was antibiotics, followed by immediate-release combination medications and non-long-acting medications.

## Conclusions/Implications

Over the 10-year period examined, an increasing proportion of dental-related incidents had physician-prescribed medications, which may be attributed to the implementation of Ontario’s pharmacare program (OHIP+) in 2018. Early access to routine and preventative dental care could become more accessible as part of Canada’s new federal dental program. Results from this AHRQ will inform better resource allocation in dental and oral health program planning, potentially reducing avoidable healthcare costs.

